# *Weissella cibaria* Relieves Gut Inflammation Caused by *Escherichia coli* through Inflammation Modulation and Gut Microbiota Regulation

**DOI:** 10.3390/foods13071133

**Published:** 2024-04-08

**Authors:** Xiaoyu Liu, Nan Jiang, Xinyue Wang, Haowen Yan, Lili Guan, Lingcong Kong, Jingrui Chen, Haipeng Zhang, Hongxia Ma

**Affiliations:** 1 College of Life Science, Jilin Agricultural University, Xincheng Street No. 2888, Changchun 130118, China; 20211259@mails.jlau.edu.cn (X.L.); jiangnan@mails.jlau.edu.cn (N.J.); wangxinyue@mails.jlau.edu.cn (X.W.); yanhaowen@mails.jlau.edu.cn (H.Y.); llguan@jlau.edu.cn (L.G.); 2 The Engineering Research Center of Bioreactor and Drug Development, Ministry of Education, Jilin Agricultural University, Xincheng Street No. 2888, Changchun 130118, China; 3 College of Veterinary Medicine, Jilin Agricultural University, Xincheng Street No. 2888, Changchun 130118, Chinachenjingrui@jlau.edu.cn (J.C.); 4 The Key Laboratory of New Veterinary Drug Research, Development of Jilin Province, Jilin Agricultural University, Xincheng Street No. 2888, Changchun 130118, China

**Keywords:** *Weissella cibaria* P-8, multi-drug-resistant *Escherichia coli* B2, inflammatory factor, intestinal flora

## Abstract

The emergence of multi-drug-resistant (MDR) pathogens has considerably challenged the development of new drugs. Probiotics that inhibit MDR pathogens offer advantages over chemical antibiotics and drugs due to their increased safety and fewer side effects. This study reported that *Weissella cibaria* P-8 isolated from pickles showed excellent antibacterial activity against intestinal pathogens, particularly the antibacterial activity against MDR *Escherichia coli* B2 was the highest. This study showed that the survival rates of *W. cibaria* P-8 at pH 2.0 and 0.3% bile salt concentration were 72% and 71.56%, respectively, and it still had antibacterial activity under pepsin, trypsin, protease K, and catalase hydrolysis. Moreover, *W. cibaria* P-8 inhibits the expression of inflammatory factors interleukin-1β, tumor necrosis factor-α, and interleukin-6, upregulates the interleukin-10 level, and increases total antioxidant capacity and superoxide dismutase enzyme activity in serum. *W. cibaria* P-8 also efficiently repairs intestinal damage caused by *E. coli* infection. The gut microbiota analysis demonstrated that *W. cibaria* P-8 colonizes the intestine and increases the abundance of some beneficial intestinal microorganisms, particularly Prevotella. In conclusion, *W. cibaria* P-8 alleviated MDR *E. coli*-induced intestinal inflammation by regulating inflammatory cytokine and enzyme activity and rebalancing the gut microbiota, which could provide the foundation for subsequent clinical analyses and probiotic product development.

## 1. Introduction

Extensive broad-spectrum antibiotic use has led to the emergence of many multi-drug-resistant (MDR) gut pathogens, particularly MDR *Escherichia coli* (*E. coli* B2). *Escherichia coli* is one of the most common bacteria in the intestine. Among *E. coli*, the strains of B2 lineage usually carry a 54 kb polyketide synthase (pks) pathogenic gene, which encodes the biosynthetic enzyme of the *E. coli* toxin colibactin [1]. *E. coli* causes serious intestinal health disorders, including changed intestinal flora, impaired barrier function, and immune system destruction, leading to diarrhea, enteritis, several host diseases, and even death [2]. This poses a serious threat to human gut health and food safety [3]. Therefore, new ways to combat MDR gut pathogens should be identified.

Probiotics are recognized by the World Health Organization as “living microorganisms” that, when given in a certain amount, promote health. The most frequently used probiotic supplements are lactic acid bacteria (LAB), including *Lactobacilli*, *Streptococci*, *Lactococcus*, *Enterococci*, and *Lactobacillus* [4]. Various analyses have demonstrated the health-promoting effects of LAB, including pathogenic bacteria inhibition, immunomodulation, microbiota community manipulation, epithelial cell proliferation and differentiation stimulation, and gut barrier regulation [5]. *Weissella* was reclassified into the family Lactobacillaceae in 1993 [6]. Recent analyses have reported that *Weissella* has probiotic and anti-inflammatory properties. For example, Sujaya et al. [7] found that *Weissella confusa* F213 attenuates clinical symptoms and inflammation and maintains mucosal integrity in induced colitis in vivo using an intestinal inflammatory infection model. El-Mekkawy et al. [8] found that *W. confusa* K3 cell-free supernatant inhibits *E. coli* U60. Bao et al. [9] found that *Weissella cibaria* protects against upper respiratory tract pathogens. However, no study has focused on the regulation of the immune system by *Weissella* and its influence on intestinal flora.

In this study, ropy *W. cibaria* strains with good antibacterial activity against MDR *E. coli* were isolated from pickles. We investigated the probiotic properties of *W. cibaria* strains, including antibacterial activity, antibiotic susceptibility, and tolerance to simulated gastrointestinal fluid and bile salts. Moreover, following the administration of different doses of *W. cibaria* in mice challenged with *E. coli* B2, inflammatory factors, enzyme activity, and intestinal flora changes were measured to clarify the mechanisms whereby *W. cibaria* strain relieves intestinal inflammation and provide a basis for the effective application of these bacteria as functional food supplements.

## 2. Materials and Methods

### 2.1. Bacterial Strain Identification

*W. cibaria* strain P-8 was isolated from pickles. Under sterile conditions, 10 mL pickled water sample was placed in a 50 mL centrifuge tube containing 25 mL normal saline, incubated at 28 °C for 24 h, and diluted by a 10-fold gradient [10]. The appropriate dilution was inoculated on MRS agar and cultured anaerobically at 28 °C for 48 h. Single white colonies were selected and purified for three generations by streaking on MRS agar. Finally, the bacteria in the culture medium were transferred to a test tube containing 10 mL MRS broth and incubated at 28 °C for 48 h, and then, cultures were stored in 80% (*w*/*v*) glycerol at −80 °C for further use [11].

### 2.2. Sequencing and Phylogenetic Analysis

16S rRNA sequencing was used to identify strain P-8. DNA was extracted using a Genomic DNA Purification Kit (Sangon Biotech, Shanghai, China), and the 16S rRNA gene was amplified with 27F (5′-AGAGTTTGATCCTGGCTCAG-3′) and 1492R (5′-TACGGCTACCTTGTTACGACTT-3′) primers. The PCR products were sequenced at Sangon Biotech (Changchun, China), and sequences were compared to the NCBI database using the nucleotide BLAST program (https://blast.ncbi.nlm.nih.gov/Blast.cgi, accessed on 8 August 2023). The phylogenetic tree was constructed using MEGA 5.1 software by the neighbor-joining algorithm and visualized using iTOL V5.

### 2.3. Characteristic Analysis of Weissella Cibaria P-8

#### 2.3.1. Growth Kinetic Curve, Antibacterial Activity, and Drug Sensitivity Test

The resuspended bacterial solution was cultured anaerobically on MRS agar at 28 °C for 24 h [12]. Then, a single colony was cultured anaerobically in MRS broth at 28 ° C until the optical density (OD) was 0.5. It was inoculated in a 300 mL MRS broth conical flask at 1% (*v*/*v*) and cultured anaerobically at 28 °C for 48 h [13]. During this period, the same amount of bacterial solution was collected every 2 h to determine the OD600 using the NanoDrop 2000 ultramicro spectrophotometer from Symefeishier Technology Company (Thermo Fisher Scientific, Waltham, MA, USA) [14]. The bacterial liquid collected at different times was centrifuged at 4 °C and 8500 rpm/min for 6 min, and the supernatant was collected. The obtained supernatant was filtered with a 0.22 μm sterile filter membrane for retention. The diameter of the inhibition zone of the supernatant was measured to determine the antibacterial activity of strain P-8 against *E. coli* B2, *Pseudomonas aeruginosa* Z1, *Salmonella* H9812, *Shigella castellani* Z1, *Acinetobacter baumannii* C1, *Staphylococcus aureus*, and methicillin-resistant *Staphylococcus aureus* (MRSA) [15]. The above indicator bacteria are all stored in the Key Laboratory of New Veterinary Drug Research and Development and Innovation, College of Animal Science and Technology (College of Veterinary Medicine), Jilin Agricultural University. This experiment was repeated thrice. After the strain P-8 was cultured for 24 h, the concentration of the bacterial solution was adjusted to 1 × 10^8^ CFU/mL, and an appropriate amount of bacterial solution was evenly coated on the MRS agar medium. After drying at room temperature for 3 min, the drug-sensitive tablets were attached to the MRS medium coated with the bacterial solution. In a 28 °C incubator, the results were observed after 24 h [16]. The above drug-sensitive papers were purchased from Hangzhou Microbial Reagent Co., Ltd. (Hangzhou, China), and the concentration of drug-sensitive papers is described in Table S1. This procedure was repeated thrice.

#### 2.3.2. Thermal and Enzymatic Stability

Proteinase K, catalase, trypsin, and pepsin (final concentration: 1 mg/mL) were added to the P-8 cell-free supernatant, incubated at 37 °C for 60 min, and treated at 100 °C for 10 min [17]. The P-8 cell-free supernatant without enzyme treatment was used as a control. After cooling to room temperature, the antimicrobial activity of the P-8 cell-free supernatant was verified by the agar diffusion method using *E. coli* B2 as the indicator bacteria [18]. The experiment was repeated three times. 

#### 2.3.3. Tolerance to Low pH

The low pH (pH = 2.0) tolerance assay was performed according to the methods used by Bamgbose et al. [19], with slight modifications. Briefly, actively growing overnight *W. cibaria* P-8 culture was harvested by centrifugation (5000× *g*, 10 min, 4 °C) and washed twice with PBS (pH 7.2). The harvested cells were reintroduced into MRS broth lowered to pH = 2 using 1 N hydrochloric acid (HCL) followed by incubation at 28 °C under an anaerobic condition. Tolerance to low pH was assessed by monitoring the OD_620_ for 3 h [20]. MRS broth (pH 6.5) with cell and cell-free suspensions served as positive and negative controls, respectively. The percentage growth rate per hour was calculated using the following formula:

$$\mathrm{Growth}\;\mathrm{rate}\;\mathrm{per}\;\mathrm{hour}\;\left(\%\right)=\left[\left(\frac{\mathrm{Final}\;O.D.}{\mathrm{Initial}\;O.D.}\right)-1\right]\times 100/\mathrm{TD}$$

Here, TD is the time difference.

#### 2.3.4. Tolerance to 0.3% Bile Salt

*W. cibaria* P-8 was cultured in MRS broth overnight at 37 °C to a stable growth phase. Then, 2% inoculum was added to MRS broth with or without 0.3% (*m*/*v*) bile salt and incubated for 3 h. The absorbance was then detected using a spectrophotometer at 600 nm [21]. The percentage growth rate per hour was calculated using the following formula:

$$\mathrm{Growth}\;\mathrm{rate}\;\mathrm{per}\;\mathrm{hour}\;\left(\%\right)=\left[\left(\frac{\mathrm{Final}\;O.D.}{\mathrm{Initial}\;O.D.}\right)-1\right]\times 100/\mathrm{TD}$$

Here, TD is the time difference.

#### 2.3.5. Adhesion of *W. cibaria* P-8 to Hydrocarbons 

Surface hydrophobicity of *W. cibaria* P-8 was determined using the microbial adhesion to hydrocarbons (MATH) method [22]. The strains were inoculated in MRS broth and incubated overnight at 28 °C. Then, the bacteria cultured to a stable growth phase were loaded into two centrifuge tubes and centrifuged at 8000× *g* for 10 min. The bacterial pellets were washed twice with sterile PBS solution (pH = 7.2) and then mixed with the PBS solution till 0.5 ± 0.05 OD_600_ (A_0_). Hydrophobic molecules tend to be nonpolar and therefore more soluble in neutral and nonpolar solutions (such as organic solvents). The strain suspension (3 mL) was vortexed, mixed evenly with 1 mL xylene, and rested for 10 min. After vortexing and shaking, the sample was rested for 30 min at 37 °C. The aqueous phase was carefully aspirated, and the OD_600_ of the aqueous phase was measured (A_1_) [23]. This test was repeated three times. The strain hydrophobicity was calculated using the following formula:

$$\mathrm{AA}\;\left(\%\right)=\left(1-\frac{A_{1}}{A_{0}}\right)\times 100\%$$

#### 2.3.6. Autoagglutination Assay

The strains were inoculated in MRS broth and incubated overnight at 28 °C. Then, the bacteria cultured to a stable growth phase were loaded into two centrifuge tubes and centrifuged at 8000× *g* for 10 min. The bacterial pellets were washed twice with sterile PBS solution (pH = 7.2) and then mixed with the PBS solution till 0.5 ± 0.05 OD_600_. The OD_600_ of the pretreated *W. cibaria* P-8 strain suspension (B_0_) was determined; the strain was shaken for 10 s and rested for 4 h, and the OD_600_ of the strain supernatant was measured (B_1_) [24]. The test was repeated three times, and the self-agglutination ability of the strain was calculated using the following formula:

$$\mathrm{Autoagglutination}\;\left(\%\right)=\left(1-\frac{B_{1}}{B_{0}}\right)\times 100\%$$

### 2.4. Oral W. cibaria P-8 Administration and Sample Collection in Mouse Study

**Figure 1:**
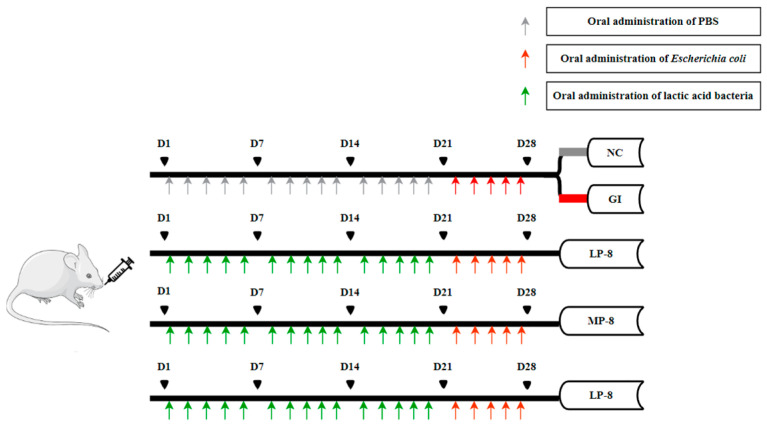
Animal model grouping.

As shown in Figure 1, 6- or 8-week-old KM mice weighing 18–22 g were purchased from Liaoning Changsheng Biological Experimental Animal Co., Ltd. (Changchun, China), and fed ad libitum. The mice were kept in a quiet and ventilated environment at 25 °C with 50% humidity and a 12/12 h light and dark cycle [25]. The mice were randomly divided into five groups and treated as follows: (1) intragastrically administered with 200 µL PBS for 4 weeks as a negative control group (NC), (2) intragastrically administered with *E. coli* B2 (2 × 10^9^ CFU) for one week to induce intestinal inflammation as a gut inflammatory control group (GI), (3) gavaged with *W. cibaria* P-8 (2 × 10^9^ CFU) for 3 weeks and then *E. coli* B2 (2 × 10^9^ CFU) for 1 week to infect the mice intestines as a low-dose group (LP-8), (4) intragastrically administered with *W. cibaria* P-8 (2 × 10^10^ CFU) for 3 weeks and then *E. coli* B2 (2 × 10^9^ CFU) for 1 week to infect the mice intestines as a medium-dose group (MP-8), and (5) intragastrically administered with *W. cibaria* P-8 (2 × 10^11^ CFU) for 3 weeks and then *E. coli* B2 (2 × 10^9^ CFU) for 1 week to infect the mice intestines as a high-dose group (HP-8). At the end of the experiment, the body weights and temperatures of the mice were recorded, and the blood and feces of all mice were collected aseptically before euthanasia [26]. The fecal samples were suspended in sterile saline to determine the live *W. cibaria* P-8 count. The laboratory animal usage license (SYXK-2018-0023) was issued by the Laboratory Animal Center of Jilin Agricultural University Changchun, China. All animal analyses (including the mice euthanasia procedure) were performed in compliance with the regulations and guidelines of Institutional Animal Care of Jilin Agricultural University Changchun, China.

### 2.5. Biochemical Assays of Serum

On day 28, the mice were fasted for 7 h and euthanized by cervical dislocation after collecting blood from the orbit [27]. Blood samples were immediately placed in a centrifuge tube, placed in a water bath at 37 °C for 1–2 h, and then stored at 4 °C for 3–4 h. The blood clot was retracted and centrifuged at 3000× *g* for 15 min, and the serum was collected as the supernatant [28]. The interleukin (IL)-1β, IL-6, IL-10, tumor necrosis factor (TNF)-α, and superoxide dismutase (SOD) levels and total antioxidant capacity (T-AOC) in the serum were determined with ELISA kits (Nanjing Jiancheng Bioengineering Institute, Nanjing, China) according to the instructions provided by the manufacturer.

### 2.6. Hematoxylin and Eosin Staining of Liver and Gut

The liver and intestinal tissues of the mice were stained with hematoxylin and eosin (H & E). Briefly, the liver, the jejunum, and specimens were immersed in 4% paraformaldehyde, waxed, and sectioned into 5 μm-thick sections [29]. The sections were dewaxed, dehydrated, immersed in graded alcohol, and stained with H & E. An Eclipse CI-L camera microscope was used to observe the target tissue for imaging purposes, with a magnification of ×100. During imaging, the tissue filled with the entire field of view as much as possible to ensure that the background light of each photo was consistent. After the imaging was completed, the image-Pro Plus 6.0 analysis software was used to unify millimeter as the standard unit.

### 2.7. Fecal Microbiota Analysis by 16S rRNA Gene Sequencing

Under stressful conditions, fecal samples were collected from three randomly selected mice of each group. Total DNA was extracted using a DNA Extraction Kit (NucleoSpin, MN, Düren, Germany) according to the manufacturer’s instructions. The V3–V4 hypervariable regions of the bacterial 16S rRNA genes were PCR-amplified and sequenced using an Illumina MiSeq PE300 platform. Operational taxonomic units (OTUs) with 97% similarity cutoff were clustered using UPARSE version 7.1 (V. 7.1; http://drive5.com/uparse/, accessed on 4 October 2023), and chimeric sequences were identified and removed. The taxonomic status of each 16S rRNA gene sequence was classified by the RDP Classifier (http://rdp.cme.msu.edu/, accessed on 16 October 2023) against the 16S rRNA database, with a 70% confidence threshold. A rarefaction curve was drawn to evaluate whether the sequencing amount was sufficient to cover all groups and indirectly reflect the richness of the species in the sample. Species richness was evaluated using the Simpson’s index. Cylindrical coordinate analysis (PCoA) was performed based on the Weighted Unifrac between samples. Indicator species (biomarker) analysis was performed using a Wayne diagram to evaluate the OTU richness. Finally, the species composition of each sample at each classification level was counted, and the change in species abundance of different samples at each classification level was visually displayed in the form of a stack diagram. All raw reads in the study were deposited into the NCBI Sequence Read Archive (SRA) database (accession #PRJNA1060486). 

### 2.8. Statistical Analysis

All analyses were performed in triplicate using the GraphPad Prism 9 software (GraphPad Software, Inc., San Diego, CA, USA). One-way analysis of variance (ANOVA) and Duncan’s test for multiple comparisons were performed using SPSS (Version 22.0) to analyze the differences among the groups, which are shown with letters.

## 3. Results

### 3.1. Strain Identification

**Figure 2:**
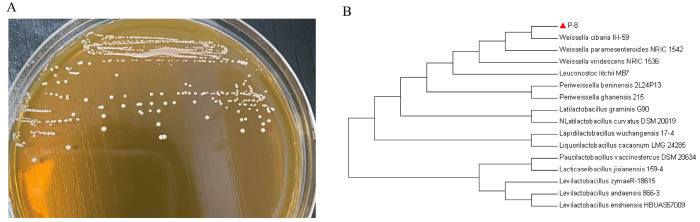
The colony morphology of strain P-8 and the phylogenetic relationship between *Weissella cibaria* P-8 and its related species based on 16S rRNA gene sequence: (**A**) colonial morphology and (**B**) phylogenetic tree.

The samples were preliminarily screened according to the above method, and the strain P-8 with a good antibacterial activity against MDR *E. coli* B2 was further analyzed. The morphological characteristics of the isolated strains were determined. Strain P-8 was a white round spherical colony with acid-producing Gram-positive cocci (Figure 2A). Phylogenetic analysis of 16S rRNA gene sequence showed that the similarity between strain P-8 and *W. cibaria* II-I-59 (Figure 2B) was 100%.

### 3.2. Characteristics of W. cibaria P-8

**Figure 3:**
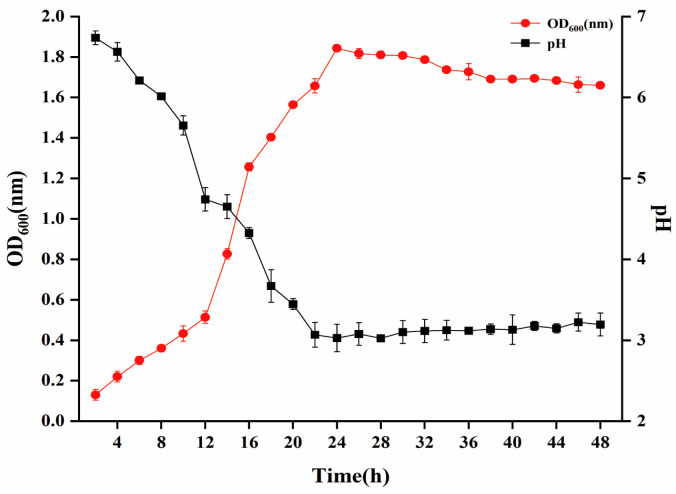
Growth curve of *Weissella cibaria* P-8 and its corresponding pH change curve.

The growth curve of *W. cibaria* P-8 (Figure 3) shows that strain P-8 grew exponentially at 4–16 h and growth plateaued at 16–24 h, and some bacteria aged and died after 26 h. The pH also changed significantly with culture time, particularly when the bacteria were in the logarithmic growth period, rapidly dropping to approximately 3.6 after 18 h, and indicating that strain P-8 produced many acidic substances at this stage. After 24 h, the pH remained constant. The antibacterial activities of strain P-8 against common pathogenic bacteria are shown in Table 1. It exhibits antibacterial activities against *E. coli*, *Staphylococcus aureus*, *Shigella*, and *Salmonella*. Among them, the antibacterial activity against *E. coli* B2 is the best (inhibition zone diameter: 24.67 ± 0.58 mm); the stability of the enzyme of strain P-8 is shown in Table S1. After protease hydrolysis, pepsin, catalase, and trypsin exhibited antibacterial activity. The inhibition zone diameter was determined for strain P-8 by the K-B disk agar diffusion method. The results showed that strain P-8 was highly sensitive to amoxicillin and florfenicol, and the sensitivity to amoxicillin was the highest, with a 33.67 ± 1.53 mm inhibition zone (Table S2). Additionally, the survival rate of strain P-8 was 72% at pH 2.0 and 71.56% in MRS broth containing 0.3% pig bile salt, while the hydrophobicity and self-aggregation ability were 82.46% and 58.24%, respectively.

### 3.3. Effects of W. cibaria P-8 in the Inflammation of Serum and Gut in Mice

**Figure 4:**
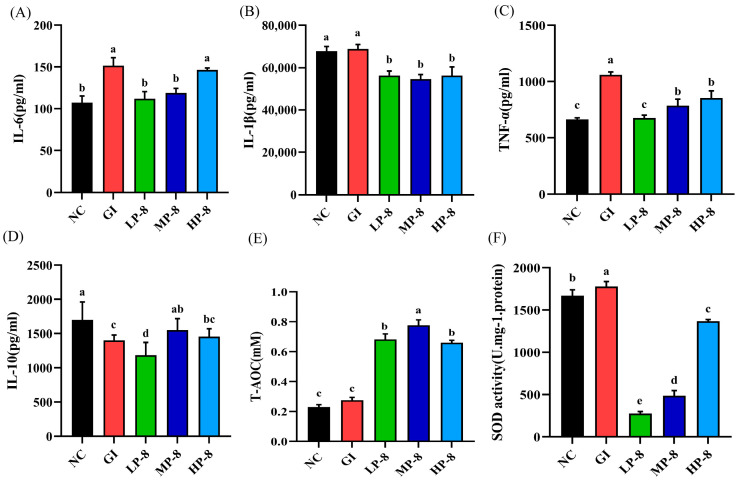
Effects of *Weissella cibaria* P-8 on serum: (**A**) IL-6, (**B**) IL-1β, (**C**) TNF-α, (**D**) SOD, (**E**) T-AOC, and (**F**) IL-10 levels of mice. Different letters in the figure indicate significant differences in levels between groups (*p* < 0.05).

To compare the immunomodulatory effects of *W. cibaria* P-8 on *E. coli* B2-infected mice, serum cytokine levels were measured by ELISA. The IL-6, IL-1β, and TNF-α levels significantly increased in the GI group than those in the NC group (Figure 4A–C). Moreover, *W. cibaria* P-8 significantly downregulated the IL-1β, TNF-α, and IL-6 levels in the treatment groups (LP-8, MP-8, and HP-8). The IL-10 levels in *E. coli*-infected mice were significantly lower than those in the NC group mice (Figure 4D). In contrast, the IL-10 levels in the LP-8, MP-8, and HP-8 group mice were significantly higher than those in the GI group mice. The results of oxidative stress in mice showed that the serum T-AOC and SOD levels were significantly enhanced in the LP-8, MP-8, and HP-8 groups (Figure 4E,F).

### 3.4. Alleviation of Liver and Gut Morphologies in Mice Fed with W. cibaria P-8 and Challenged with E. coli B2

**Figure 5:**
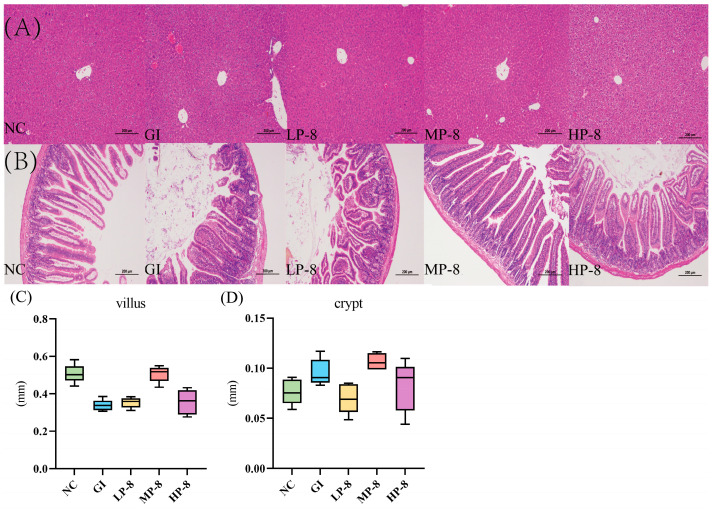
Histopathological analysis of the liver and the jejunum in mice (HE staining, ×100): (**A**) the liver, (**B**) the jejunum, (**C**) the villus height, and (**D**) the crypt depth.

The H & E staining result showed liver vacuolization in GI mice, while *W. cibaria* P-8 attenuated liver vacuolization in the LP-8, MP-8, and HP-8 group mice (Figure 5A). The jejunal morphologies of the NC, GI, LP-8, MP-8, and HP-8 group mice were also evaluated (Figure 5B). The GI tracts of the infected mice showed inflammatory infiltration and the villus height and crypt depth decreased and increased, respectively, than those in the NC group mice. The villus height and jejunal crypt depth of the LP-8 and HP-8 group mice did not change significantly than those of the GI group mice, while those of the MP-8 group mice increased by 506.043 ± 0.043 and 106.684 ± 0.0083 μm, respectively (Figure 5C,D).

### 3.5. Colonization of W. cibaria P-8 in the Gut and the Composition of Gut Microbiota in Mice

**Figure 6:**
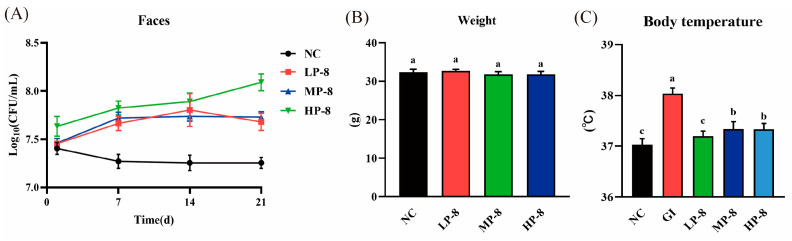
(**A**) Counts of lactic acid bacteria (LAB) in feces of *Weissella cibaria* P-8-fed (LP-8, MP-8, HP-8), GI, and NC group mice at days 1, 7, 14, and 21. (**B**) Body weight. (**C**)Temperature. Different letters in the figure indicate significant differences in levels between groups (*p* < 0.05).

**Figure 7:**
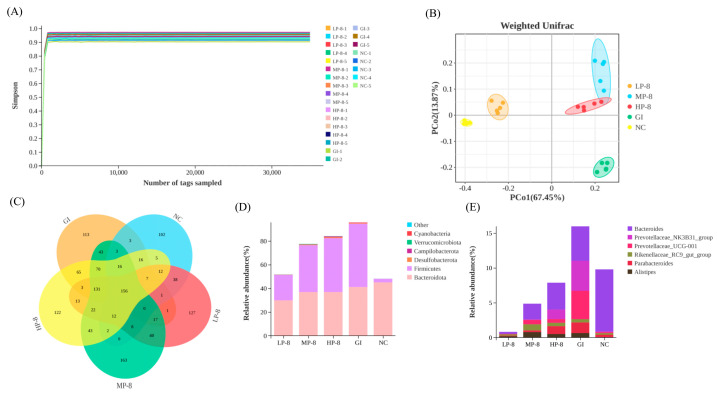
Gut microbiota profiles in mice fed with *Weissella cibaria* P-8 strains and challenged with *Escherichia coli*: (**A**) Shannon–Wiener curve analysis of 16S rRNA, (**B**) Venn diagram of ASV/OTU in the feces, (**C**) principal coordinate analysis (PCoA) on the weighted UniFrac distance matrix of gut microbiota between NC, GI, LP-8, MP-8, and HP-8 groups, (**D**) bar graphs showing the relative abundance of different bacteria at the genus level and the gate level, and (**E**) bar graphs showing the relative abundance of different bacteria at the genus level and the gate level.

In mice fed with *W. cibaria* P-8, feces were collected and cultured to determine the number of viable *W. cibaria* P-8 in the intestine. The P-8 count in the LP-8, MP-8, and HP-8 group mice were 8.12 ± 0.30, 8.17 ± 0.2, and 8.38 ± 0.13 Log_10_ CFU/mL, respectively, it was and significantly higher than that in the NC group mice (7.13 ± 0.04 Log_10_ CFU/mL; Figure 6A). No significant weight gain was observed in the LP-8, MP-8, HP-8, or NC group mice during the study (Figure 6B). In addition, the body temperature of the LP-8, MP-8, and HP-8 group mice showed a downward trend than that of the GI group mice (Figure 6C). To investigate the effect of strain P-8 on the intestinal microorganisms in mice, the microbiota of mouse feces was analyzed using MiSeq 16S rRNA sequencing. As shown in Figure 6, the sequencing depth of the 16S rRNA region and the Shannon–Wiener curve appeared to have reached an asymptote, indicating that sufficient 16S rRNA gene sequences (Figure 7A) were captured from mouse feces using the selected primer set. Specifically, the number of OTUs in the LP-8, MP-8, and HP-8 group mice was significantly higher than that in the NC and GI group mice, and the number of OTUs was the highest in the MP-8 group mice (Figure 7B). Principal coordinate analysis (PCoA) showed that *W. cibaria* P-8 shaped the gut microbiota of *E. coli* B2-infected mice and regulated them to a normal microbial community (Figure 7C). The distribution of phylum- and genus-level bacterial diversity (Figure 7D) was determined by the 16S RNA amplicon sequencing of each sample from the five analyzed groups. Analysis of bacterial diversity at the phylum level showed that the number of OTUs similar to *Firmicutes* and *Bacteroidetes* decreased significantly compared to the GI group mice. Analysis of bacterial diversity at the genus level (Figure 7E) showed that the number of OTUs similar to *Bacteroides* and *Prevotella* decreased significantly in the LP-8, MP-8, and HP-8 group mice than that in the GI group mice. Moreover, the number of OTUs similar to the genus Parabacteroides in the LP-8, MP-8, and HP-8 group mice significantly reduced and increased than that in the GI and NC group mice, respectively.

## 4. Discussion

As intestinal pathogens have shown increased drug resistance, gastrointestinal discomfort and liver damage in humans and animals has been a hot topic. Probiotics not only provide resistance to gastrointestinal pathogens and improve immunity but also improve the gastrointestinal flora [30]. In this study, *W. cibaria* P-8 showed excellent inhibition against *Pseudomonas aeruginosa* Z1, *Salmonella* H9812, *Acinetobacter baumannii* C1, and MRSA, with the highest inhibitory activity against *E. coli* B2. In addition, *W. cibaria* P-8 showed good hydrophobicity and self-aggregation. The activity and survival rate of *W. cibaria* P-8 are some of the most important parameters for evaluating its ability as a good probiotic in vivo [31]. Lactic acid bacteria form a stable biological barrier by adhering to the intestinal mucosa and have important physiological functions such as inhibiting the invasion and proliferation of pathogenic bacteria. Therefore, adhesion ability is one of the important indicators to evaluate the probiotic characteristics of lactic acid bacteria [32]. Studies have shown that the surface hydrophobicity of bacteria is not only related to the adhesion of bacteria to the host but also to the removal of pathogenic bacteria [33]. The results of hydrophobicity and self-agglutination of strain p-8 showed that the hydrophobicity of *W. cibaria* P-8 reached 82.46% and the self-agglutination was 58.24% after treatment; thus, it had good hydrophobicity and self-agglutination. This indicates that it can colonize in the gastrointestinal tract and exert probiotic functions. Probiotics need to survive in the small intestine through the acidic environment of the stomach and the alkaline environment of the colon to resist bile salts [34]. When the pH of Gastric juice is about 3.0, gastric digestion could last for 3 h [34]. Studies have reported that the physiological concentration of human bile is between 0.3% and 0.5%, the time of food passing through the small intestine is about 4 h, and the average concentration of bile is 0.3% [35]. The feasibility of the experimental strain as a probiotic was evaluated by simulating the gastrointestinal environment, comparing the low pH environment and the high bile concentration environment. In this study, pH 2.0 and 0.3% bile salt concentration were selected as treatment conditions. The survival rate of *W. cibaria* P-8 was 72% after pH 2.0 treatment for 3 h, and the survival rate was 71.56% under 0.3% bile salt concentration treatment. The results showed that *W. cibaria* P-8 had the ability to grow and survive under low pH and 0.3% bile salt conditions, indicating that the strain could tolerate gastrointestinal environmental conditions.

The excessive expression of multiple cytokines in the inflamed intestine is the main factor of intestinal injury [36]. Probiotics also play a beneficial role in alleviating intestinal inflammation [37]. Cytokines are molecular messengers that can regulate and mediate immune responses, including pro-inflammatory factors and anti-inflammatory factors. Through their role, the body can fight infection and other pathological conditions [38]. In this study, we found that *W. cibaria* P-8 treatment can effectively change the intestinal morphology and tight junctions, upregulate pro-inflammatory cytokines, and downregulate anti-inflammatory cytokines in the intestine. Serum cytokine analysis showed that LP-8 reduced the levels of inflammatory factors IL-6 and TNF-α, and LP-8, MP-8, and HP-8 treatment had no significant effect on the pro-inflammatory factor IL-1β. Cao et al. found that the level of pro-inflammatory factors in infected mice treated with lactic acid bacteria XII decreased, which was consistent with the results of this study [39]. The MP-8 treatment group significantly increased the level of anti-inflammatory factor IL-10, and Na et al. found that lactic acid bacteria isolated from breast milk had a good therapeutic effect on the food-borne infection of multidrug-resistant *E. coli* in mice. After treatment, the level of anti-inflammatory factors in cells increased, which was consistent with the results of this study [40]. T-AOC is used to evaluate the antioxidant capacity of bioactive substances [41]. SOD is an antioxidant metal enzyme existing in organisms [42]. It can catalyze the disproportionation of superoxide anion radicals to produce oxygen and hydrogen peroxide and plays a vital role in the balance of oxidation and antioxidation in the body. The T-AOC and SOD of lactic acid bacteria are important indicators for their treatment and colonization as probiotics. The analysis showed that the MP-8 treatment group increased the T-AOC level, and the MP-8 treatment group increased the SOD level. Zhong et al. found that, after *E. coli* infection, the T-AOC and SDO activity levels of the treatment group fed with mixed lactic acid bacteria increased, which was consistent with the results of this study [11]. *Weissella* strain JW15 isolated from pickles by Park et al. has immunostimulatory activity in vitro and can regulate the immunity of mice after oral administration [43].

To explore *W. cibaria* P-8 colonization in the gut of mice, the total viable *W. cibaria* P-8 count was determined. The viable cell counts in the LP-8, MP-8, and HP-8 group mice were higher than those in the NC group mice, indicating that *W. cibaria* P-8 can smoothly pass through the stomach after an intragastric administration and enter the intestine as a live bacterium [44]. In addition, the fecal DNA of mice sequencing after *W. cibaria* P-8 administration verified *W. cibaria* P-8 colonization. Probiotic strains stimulate the migration of mouse intestinal cells [45]. In our study, *W. cibaria* P-8-administered mice had a higher crypt depth and lesser liver vacuolation than the *E. coli* B2-infected mice, further indicating that *W. cibaria* P-8 can colonize and repair intestinal damage.

In this study, the effect of *W. cibaria* P-8 on intestinal flora in inflammatory mice was also investigated. We found that *W. cibaria* P-8 remodeled and restored the gut microbiota in mice [46]. Compared to that of the GI group mice, the intestinal microflora of LP-8, MP-8, and HP-8 group mice was restored, and the intestinal microbial diversity improved. This is consistent with the findings of Lv et al. [47] who reported that the loss of microbial flora diversity is an important characteristic of floral imbalance. The *Weissella* strain WIKIM28 isolated from Korean mustard leaf fermented vegetable products by Lim et al. inhibited atopic dermatitis in mice. The oral administration of *W. cibaria* WIKIM28 can reduce the level of inflammatory factors in mice and reduce atopic dermatitis-like lesions in mice [48]. In addition, the diversity of the microbiota can be used to predict cell activation and dynamic balance of the gut microbiota [39]. At the phylum level, *W. cibaria* P-8 gavage reduced the abundance of Bacteroidetes and Firmicutes in *E. coli* B2-infected mice. This highlights the diversity of probiotic supplementation in improve intestinal health [49]. At the genus level, the richness of LAB in the treatment group mice increased compared to that in the other mice group. In particular, the abundance of beneficial intestinal microorganisms, particularly Prevotella, increased after *W. cibaria* P-8 treatment [50]. Prevotella is generally considered a healthy plant-based diet-associated bacterium that acts as a probiotic in the human body [51]. Importantly, the gut microbiota and inflammatory cytokines in inflammatory pathways are strongly correlated. This finding is consistent with the phenomenon observed in our study. These results suggest that *W. cibaria* P-8 may regulate intestinal immune homeostasis by regulating the intestinal microbiota in mice.

## 5. Conclusions

In summary, *W. cibaria* P-8 isolated from pickles has a good antibacterial activity against MDR *E. coli* B2 and has good bile salt tolerance and self-agglutination ability in vitro. In the mouse inflammation model, it can regulate serum biochemical indicators, restore organ damage, and reduce inflammatory response. *W. cibaria* P-8 can also regulate the expression of inflammatory factors and antioxidant capacity to normal levels. In addition, *W. cibaria* P-8 may play a role in restoring the structure and function of intestinal microbial communities. These findings suggest that it may be developed as a probiotic to improve inflammation in mice infected with MDR *E. coli* B2.

## Figures and Tables

**Table 1:** Antimicrobial spectrum of *Weissella cibaria* P-8 cell-free supernatant.

Gram Reaction and Strains	Source	Is It Pathogenic	Inhibitory Circle Diameter (mm)
**Gram-negative bacteria**			
*Escherichia coli* B2	Type strain	Yes	24.67 ± 0.58
*Pseudomonas aeruginosa* Z1	Lung of cattle	Yes	21.33 ± 0.58
*Salmonella* H9812	Intestine of cattle	Yes	20.33 ± 0.58
*Shigella castellani* Z1	Intestine of cattle	Yes	7.33 ± 0.58
*Acinetobacter baumannii* C1	Intestine of chicken	Yes	15.33 ± 0.58
**Gram-positive bacteria**			
*Staphylococcus aureus* ATCC25923	Standard strain	Yes	15.33 ± 0.58
*Methicillin-resistant Staphylococcus*	Feces of cattle	Yes	12.67 ± 0.58
*aureus* (MRSA)			

## Data Availability

The original contributions presented in the study are included in the article and Supplementary Material, further inquiries can be directed to the corresponding authors.

## Supplementary Materials

The following supporting information can be downloaded at: https://www.mdpi.com/article/10.3390/foods13071133/s1, Table S1: Enzymatic stability of *Weissella cibaria* P-8 cell-free supernatant; Table S2: Drug resistance sensitivity of *Weissella cibaria* P-8 cell-free supernatant.
