# Supplementary material for: Weissella cibaria Relieves Gut Inflammation Caused by Escherichia coli through Inflammation Modulation and Gut Microbiota Regulation
Source: Foods. 2024 Apr 8;13(7):1133. doi: 10.3390/foods13071133 (PMC11011356; doi:10.3390/foods13071133)
Supplement: Supplementary file 1 [file foods-13-01133-s001.zip › foods-2917215-supplementary.pdf]

foods

Article

Weissella cibaria Relieves Gut Inflammation Caused by *Escherichia coli* through Inflammation Modulation and Gut Microbiota Regulation

Xiaoyu Liu [1,2], Nan Jiang [1,2], Xinyue Wang [1,2], Haowen Yan [1,2], Lili Guan [1,2], Lingcong Kong [3,4], Jingrui Chen [3], Haipeng Zhang [1,2,*] and Hongxia Ma [1,2,3,*]

Supplementary Table

Table S1. Enzymatic stability of *Weissella cibaria* P-8 cell-free supernatant.

Enzyme	Producer	Broth medium	Inhibitory circle diameter (mm)
Catalase	Maclean	LB	22.67±0.28
Trypsin	Maclean	LB	21.46±0.34
Pepsin	Maclean	LB	22.33±0.12
Proteinase k	Maclean	LB	23.52±0.19

Table S2. Drug resistance sensitivity of *Weissella cibaria* P-8 cell-free supernatant.

Category	Antibacterial drug	Drug sensitivity paper concentration (μg/piece)	Criterion for judgement			Drug sensitivity results
			esistance(R)	resonance(I)	sensitivity(S)	
β-lactams	Cefoperazone	75	≤17	18~20	≥21	I
	ceftriaxone	30	≤14	15~17	≥18	I
Aminoglycosides	gentamycin	10	≤12	13~14	≥15	R
	amikacin	30	≤14	15~16	≥17	R
macrolides	erythromycin	15	≤13	14~17	≥18	I
	azithromycin	15	≤13	14~17	≥18	I
Tetracyclines	tetracycline	30	≤14	15~18	≥19	S
	minocycline	30	≤12	13~15	≥16	S
4-quinolones	levofloxacin	5	≤12	13~17	≥18	R
	ciprofloxacin	5	≤14	15~21	≥22	S
